# Supplementary material for: The modular organization of human anatomical brain networks: Accounting for the cost of wiring
Source: Netw Neurosci. 2017 Feb 1;1(1):42–68. doi: 10.1162/NETN_a_00002 (PMC6372290; doi:10.1162/NETN_a_00002)
Supplement: Supplementary file 1 [file netn-01-42-s001.pdf]

Supplementary Materials for: "The modular organization of human anatomical brain networks: Accounting for the cost of wiring"

Richard F. Betzel,[1] John D. Medaglia[1,2], Lia Papadopoulos[3], Graham Baum[4], Ruben Gur[4], Raquel Gur[4], David Roalf[4], Theodore D. Satterthwaite[4], and Danielle S. Bassett[1,5*]

[1]Department of Bioengineering, University of Pennsylvania,
[2]Department of Psychology, University of Pennsylvania
[3]Department of Physics, University of Pennsylvania, Philadelphia, PA, 19104
[4]Neuropsychiatry Section, Department of Psychiatry, University of Pennsylvania
[5]Department of Electrical and Systems Engineering, University of Pennsylvania
Philadelphia, PA, 19104, USA

*To whom correspondence should be addressed; E-mail: dsb@seas.upenn.edu.

Contents of this Supplementary Document

This file includes the following content:

1. Supplementary Materials and Methods

2. Figures S1 to S9

3. References

1 Supplementary Materials and Methods

In the main text we presented an analysis of the modular organization of human structural brain networks. Specifically, we proposed a modification of the well-known modularity quality function (*1*), wherein we replace the null connectivity model with one that depends upon the brain's spatial embedding. In this supplement we present a number of additional analyses that demonstrate the robustness of our results.

1.1 Including *versus* excluding subcortical regions

In the main text we focused on a network composed of $N = 1014$ brain regions. These regions were based on a subdivision of the so-called Desikan-Killany atlas (*2*). That atlas consists of 68 cortical regions and 14 subcortical regions. The subdivision was constructed by dividing the 68 cortical regions into 1000 approximately equal volume regions (*3*) while not sub-dividing subcortical regions at all. As a consequence, the 1000 cortical regions tend to be of smaller volume than the 14 subcortical regions. In general, regions with larger volume will tend to have higher degree which can bias the topology of the resulting network. To avoid these biases a number of recent studies using the same parcellation have opted to exclude all sub-cortical regions from analysis (*4–7*). Here, we demonstrate that the optimal consensus modules we

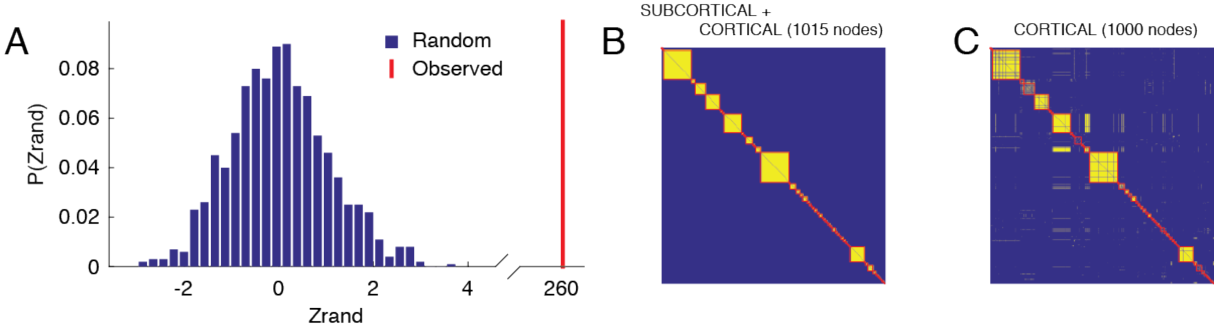

Figure S1: **Comparison of communities with and without subcortical regions**. To test whether the inclusion of subcortical regions influenced detected modules, we constructed a group-representative network comprised of $N = 1000$ cortical regions and performed modularity maximization using the SPTL model on this network. (*A*) We found that the optimal cortical partition was detected at $\gamma = 2.8$. Comparing the optimal cortical and subcortical + cortical partitions, we observed that they were highly similar to one another ($z_{rand} = 260.26$; maximum value of null distribution $z_{rand} = 3.40$, 10000 permutations). (*B*) Subcortical + cortical parcellation association matrix ordered by optimal subcortical consensus partition. (*C*) Cortical parcellation association matrix ordered by optimal subcortical consensus partition.

describe in the main text are relatively robust to our decision of whether to include or exclude subcortical regions in the network.

In parallel to our analysis presented in the main text, we generated and analyzed a group-representative connectivity matrix comprised of only the $N = 1000$ cortical regions. To differentiate this matrix from the one in the main text, we refer to the network analyzed in the main text as SUBCORTICAL + CORTICAL and the cortical only network as CORTICAL. Our analysis of the CORTICAL network was performed in precisely the same manner as our analysis of the SUBCORTICAL + CORTICAL network – e.g. we selected the optimal resolution parameter value based on the median partition similarity and we constructed a consensus partition at that parameter value. For the CORTICAL network we observed an optimal resolution parameter of $\gamma = 2.8$ (the SUBCORTICAL + CORTICAL network described in the main text achieved its optimal resolution parameter at $\gamma = 2.6$). To assess the similarity of the CORTI-

CAL and SUBCORTICAL + CORTICAL consensus partitions, we compared them using the z-score of the Rand coefficient (*8*). Specifically, we removed the sub-cortical regions from the SUBCORTICAL + CORTICAL partition so that both partitions contained the same 1000 nodes, calculated their similarity, and compared this value to a null distribution obtained by randomly and uniformly permuting the module assignments of the CORTICAL partition 10000 times. The observed similarity of the two partitions was $z_{rand} = 260.26$ while the largest similarity in the null distribution was $z_{rand} = 3.40$ (Fig. S1A). This suggests that the two partitions are similar to each other well beyond what would be expected under this specific null model. We support this statistical analysis qualitatively by showing both CORTICAL and SUBCORTICAL + CORTICAL association matrices side by side with their rows and columns ordered such that nodes assigned to the same module in the SUBCORTICAL + CORTICAL partition were positioned next to each other (Fig. S1B,C).

1.2 Robustness to choice of resolution parameter

Multi-scale modularity maximization involves modifying the modularity quality function by including the resolution parameter, γ (*9*). This modification makes it possible to tune γ to different values and uncover communities of different sizes, thereby partially mitigating the effect of the so-called "resolution limit" (*10*). Unfortunately, there is no agreed upon method for selecting the optimal value of γ; most studies avoid the issue by setting its value to its default setting of $\gamma = 1$ or by using some heuristic rule for choosing its value. In the main text we selected γ to be the value at which the detected partitions were most similar to one another (as measured by the z-score of the Rand coefficient). This resulted in us focusing on communities detected at $\gamma = 2.6$.

It is possible that different heuristics could highlight different resolution parameters. While it is beyond the scope of this paper to systematically compare such heuristics, we wish to

demonstrate that our consensus modules are robust to reasonable variations in γ. Accordingly, we compared consensus partitions obtained at $\gamma = 2.2, \ldots, 3.0$ in increments of 0.1 (nine total values) with those described in the main text. As in the previous section, our comparison involved calculating the z-score of the Rand coefficent and comparing it against a null distribution obtained through permutation testing. In general, we found that all of the consensus partitions detected in this range were much more similar to those detected at $\gamma = 2.6$ than would be expected under the null model (the minimum observed z-score over any of the nine γ values was 222.66 while the maximum value obtained in any of the null distributions was 5.43) (Fig. S2).

In addition to this statistical analysis, we show the association matrices calculated from the consensus modules detected over this same range. To facilitate comparision, we order their rows and columns according to the consensus partition obtained at $\gamma = 2.6$ (described in the main text). We see that, qualitatively, the partitions are excellent matches and that most non-zero elements are located within modules (the blocks along the diagonal) (Fig. S3A). We also show, for the same values of γ, the association matrices calculated from the "raw partitions" (i.e. those obtained from modularity maximization but before consensus clustering). (Fig. S3B).

1.3 Robustness to variation in max curvature angle

Reconstructing connectivity matrices from diffusion imaging data with tractography algorithms is challenging and prone to both false positives and false negatives (*11, 12*). In addition, tractography algorithms include multiple parameters that can influence the resulting matrices. One important parameter is the max curvature angle that determines the largest orientation change that a streamline can exhibit between integration steps before it is terminated. While it is beyond the scope of the present study to systematically test all possible values of this parameter (and others), we nonetheless wished to demonstrate that our results are consistent if we vary its value within some reasonable range.

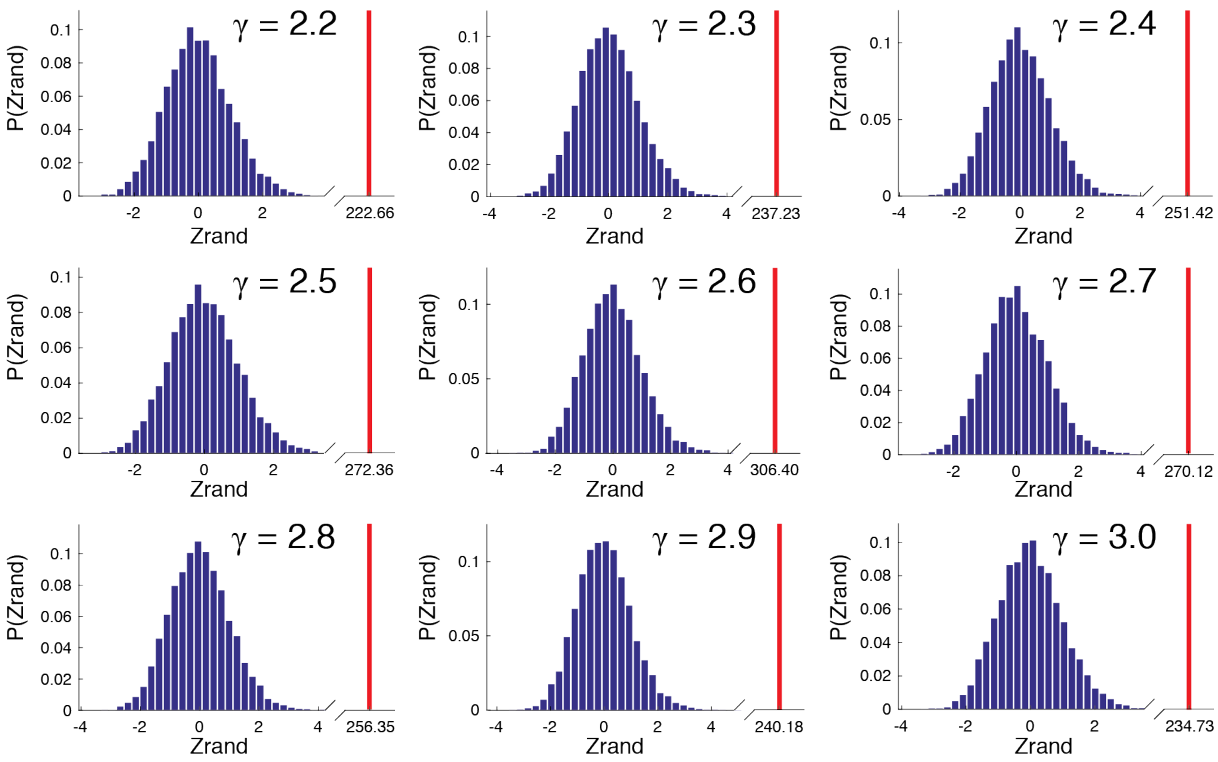

Figure S2: **Statistical assessment of community robustness to variation in** γ. We tested how similar consensus partitions from $\gamma = 2.2$ to $\gamma = 3.0$ were to the partition obtained at $\gamma = 2.6$. We calculated the similarity (z-score of the Rand coefficient) of each consensus partition with respect to the partition obtained $\gamma = 2.6$ and then generated a null distribution by calculating a comparable similarity score but where the community labels in each consensus partition were uniformly permuted at random.

The subject-level networks described in the main text were reconstructed using a max curvature angle of 35°. Accordingly, we repeated our analysis using networks reconstructed with max curvature angles of 30° and 40°. From these networks, we followed the methods described in the main text to construct group-representative networks. As, perhaps, was expected, the group-representative networks varied in terms of gross network statistics. For example, the total number of connections increased monotonically with maximum curvature angle: 51994, 80513, and 119204 connections for the 30°, 35°, and 40° cutoffs, respectively.

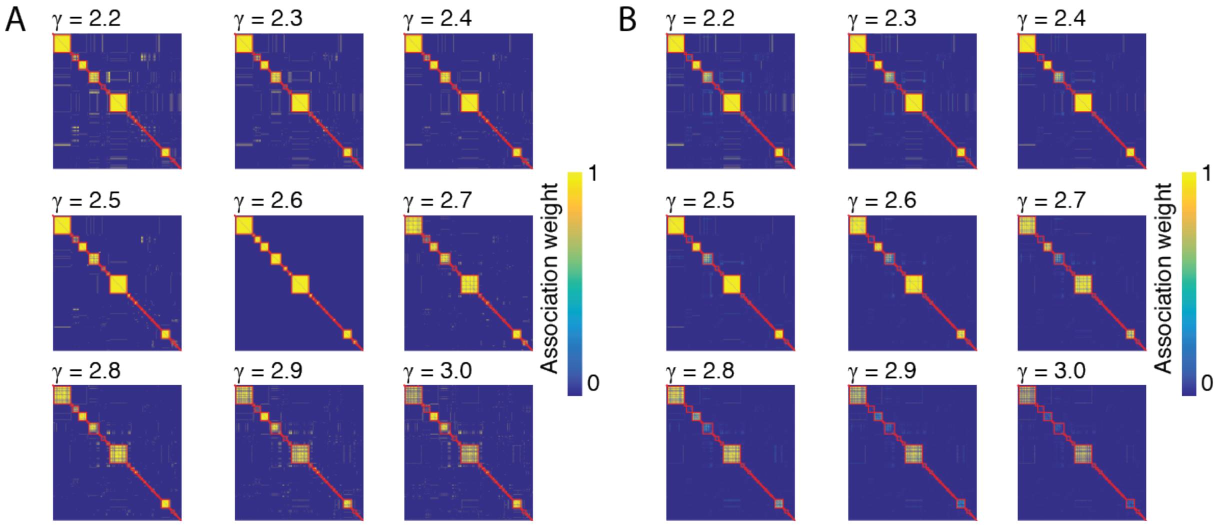

Figure S3: **Qualitative assessment of community robustness to variation in** γ. As a qualitative assessment of similarity, we show association matrices over the same range of γ constructed from consensus partitions and the ensemble of partitions obtained from the initial modularity maximization. (*A*) Association matrices constructed from consensus partitions. (*B*) Association matrices constructed from the initial modularity maximization.

Our analysis of these networks was identical to what was described in the main text. Specifically, we maximized the SPTL modularity over a range of resolution parameters and identified the optimal parameter as the one that maximized the mean pairwise similarity of partitions detected at that value. We found that for the 30° and 40° cutoffs, the optimal resolution parameters were $\gamma = 3.0$ and $\gamma = 2.1$, respectively. Once the optimal parameter was selected, we used the consensus clustering procedure to generate consensus modules.

We then calculated the similarity of these new consensus modules with the consensus modules described in the main text (where similarity was measuresd as the z-score of the Rand coefficient). To contextualize these scores, we compared them against a null distribution obtained by randomly and uniformly permuting module assignments. The observed similarity of the consensus modules obtained with maximum curvature angles of 30° and 40° to the con-

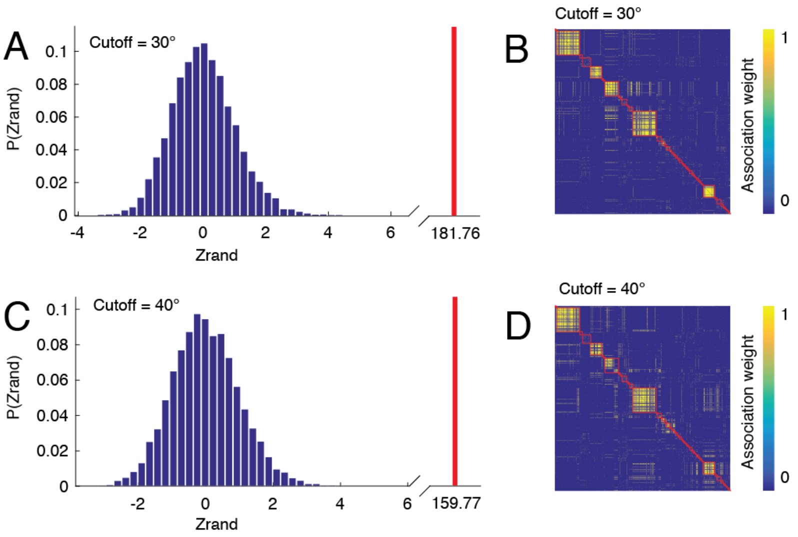

Figure S4: **Comparison of consensus modules obtained using different maximum curvature cutoffs**. (*A,C*) The z-scores of Rand coefficents of consensus modules described in the main text with consensus modules obtained using different tractography parameters – specifically maximum curvature angles of 30° and 40°. We compare a null distribution (*blue*) versus what was observed (*red*), noting that in both cases the observed value exceeds the maximum values of the null distribution by a wide margin. (*B,D*) Association matrices for consensus modules obtained using 30° and 40° maximum curvature angles. The rows and columns of each matrix have been ordered according to the consensus modules described in the main text. Note that most non-zero elements tend to fall within modules, indicating high similarity.

sensus modules described in the main text were 181.76 and 159.77, respectively, which far exceeded the maximum values obtained in either null distribution (4.48 and 4.28) (Fig. S4A,C). Based on these observations, we conclude that the consensus modules obtained using 30° and 40° cutoffs are, at least in this specific statistical sense, similar to those described in the main text. For qualitative assurance that the modules were similar to one another, we also generated association matrices for the consensus modules obtained using 30° and 40° cutoffs and ordered their rows and columns according to the module assignments described in the main text (obtained using 35° cutoff). Our expectation was that most non-zero elements would fall within modules, whereas few non-zero elements would appear between modules. Indeed, this is the

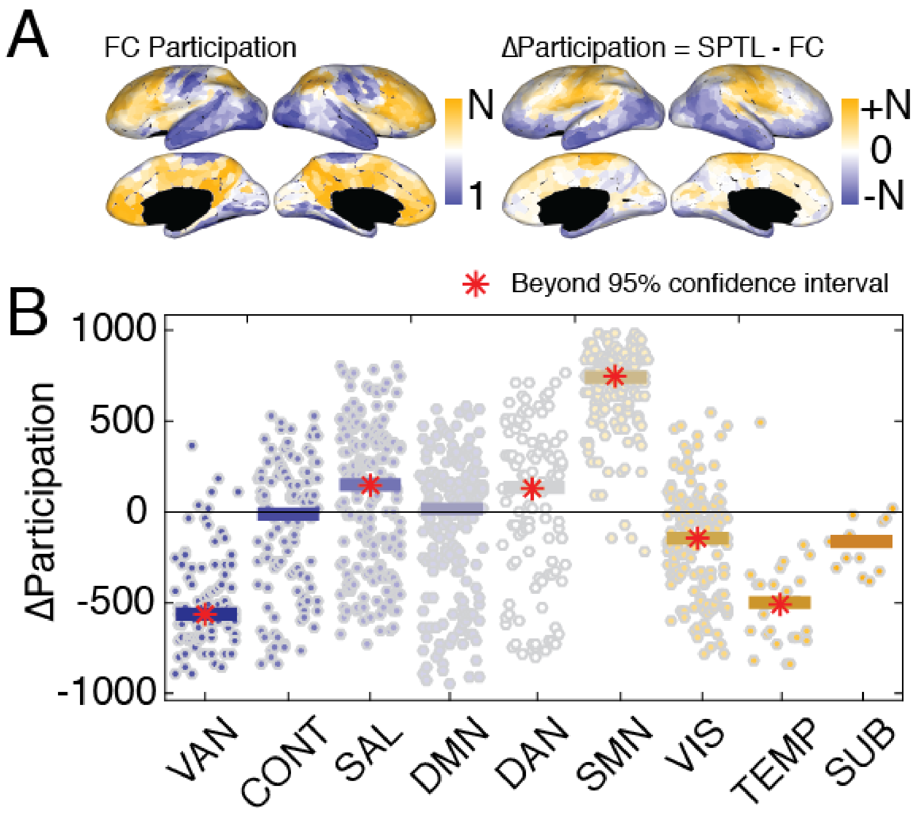

Figure S5: **Comparison of participation coefficients**. (*A*) Ranked participation coefficients obtained using a functional connectivity (FC) partition (from (*6*)). (*B*) Difference in ranked participation coefficient obtained from the SPTL model and the FC partition. (*C*) Regional participation coefficients grouped by functional system.

case (Fig. S4B,D).

1.4 Participation coefficients of structural *versus* functional partitions

In the main text we described brain regions' participation coefficients with respect to *structural partitions* obtained from structural connectivity networks. Specifically, we compared structural partitions obtained by maximizing the standard Newman-Girvan modularity with those obtained using a novel space-dependent modularity. This approach – hub classificiation based

on structural partitions – has been described in a number of high profile studies and we sought to continue this tradition (*13, 14*). However, a number of other studies have calculated participation coefficients with respect to a *functional partition* obtained, for example, from an ICA analysis of fMRI BOLD time series (*15*) or from community structure analysis of functional connectivity brain networks (*16*). This second approach makes it possible to assess indirectly the roles of individual nodes with respect to the brain's functional systems.

Therefore, in the interest of completeness, we compared participation coefficients calculated using the consensus partition obtained from the SPTL model with those obtained using the functional partition described in the main text (taken from (*6*)). We followed precisely the same methods as described in the main text: (1) we ranked participation coefficients, (2) subtracted one set of coefficients from the other, (3) and grouped these coefficients by functional system to estimate the mean change difference in participation coefficient for each system (the statistical significance of which we assessed using a permutation test). We also produced surface plots to show the spatial distribution of participation coefficients obtained using the functional partition. Though the results of this procedure are not in precise agreement with those described in the main text, they are qualitatively quite similar, serving to highlight the same changes in participation. Notably, both comparisons highlight the somatomotor network as exhibiting increased participation.

2 Additional Supplementary Figures

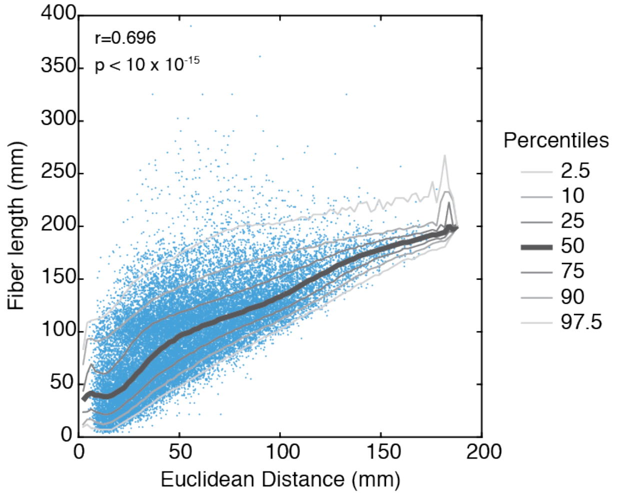

Figure S6: **Correlation of fiber length and Euclidean distance** We show a strong correlation of a connection's Euclidean distance (straight line distance between its endpoints) and its fiber length (curvilinear trajectory through space).

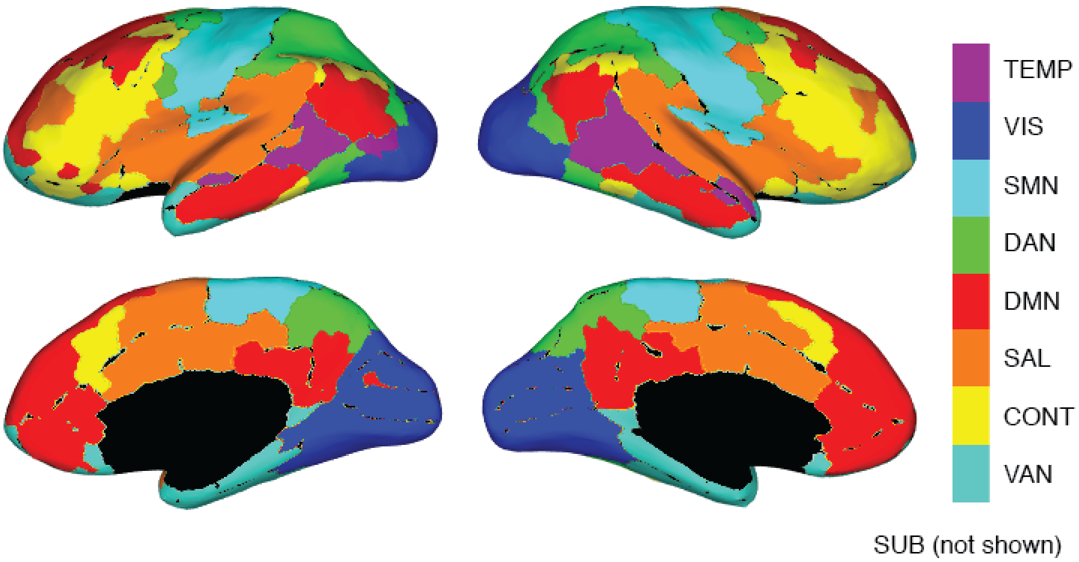

Figure S7: **Functional systems** Topographic representation of 8/9 functional systems; the subcortical system (SUB) is not shown in this rendering. The systems shown are: temporal (TEMP), visual (VIS), somatomotor (SMN), dorsal attention (DAN), default mode (DMN), salience (SAL), control (CONT), and ventral attention (VAN) networks.

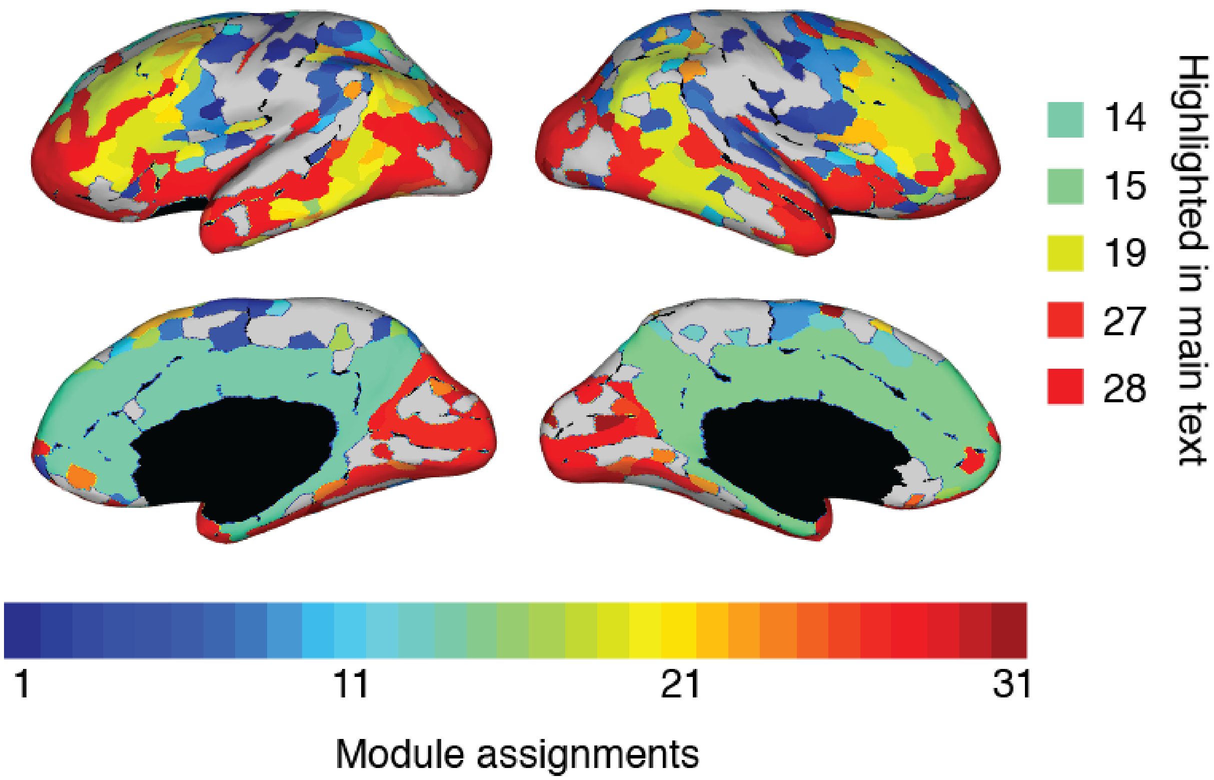

Figure S8: **All detected consensus SPTL modules for DSI dataset** Topographic representation of the 31 consensus modules detected by applying modularity maximization using the SPTL null model to the human DSI datset.

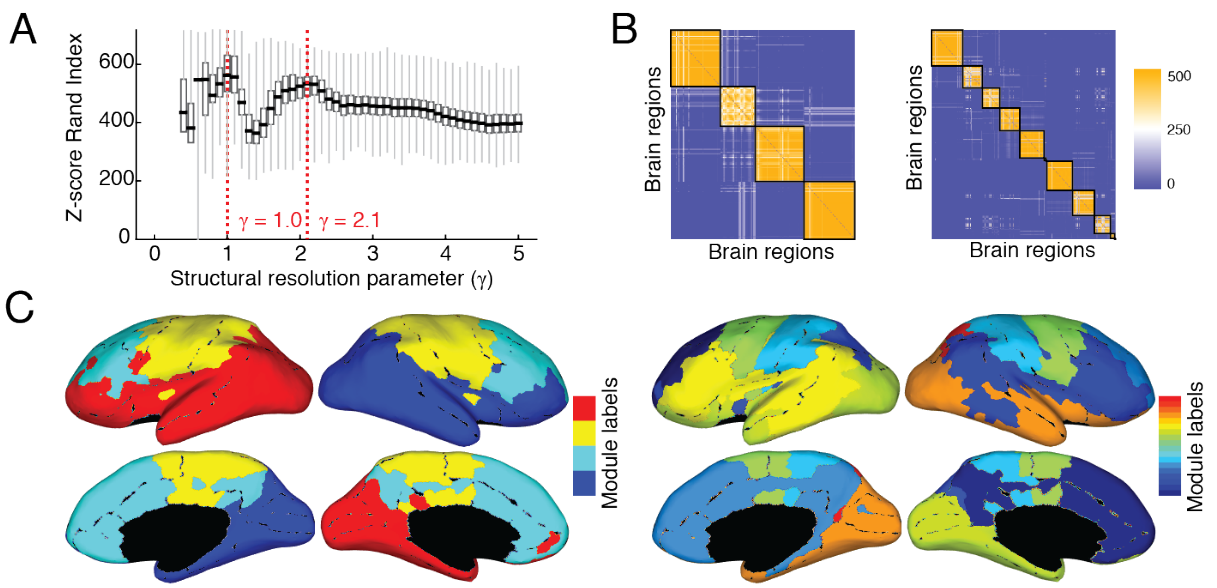

Figure S9: **NG modules in Human DSI** (*A*) Distribution of the *z*-score of the Rand indices as a function of γ. (*B*) Association matrices (fraction of times out of 500 Louvain runs that each pair of nodes were assigned to the same module) clustered according to consensus modules for the two peaks in the curve shown in panel *A*, corresponding to $\gamma = 1.0$ (*left*) and $\gamma = 2.1$ (*right*). (*C*) Module assignments on the cortical surface for modules detected at $\gamma = 1.0$ (*left*) and $\gamma = 2.1$ (*right*).

References and Notes

1. M. E. Newman, *Proceedings of the national academy of sciences* **103**, 8577 (2006).

2. R. S. Desikan, *et al.*, *Neuroimage* **31**, 968 (2006).

3. L. Cammoun, *et al.*, *Journal of neuroscience methods* **203**, 386 (2012).

4. J. Goñi, *et al.*, *Proceedings of the National Academy of Sciences* **111**, 833 (2014).

5. R. F. Betzel, *et al.*, *Network Science* **1**, 353 (2013).

6. B. Mišić, *et al.*, *Neuron* **86**, 1518 (2015).

7. A. Avena-Koenigsberger, *et al.*, *Phil. Trans. R. Soc. B* **369**, 20130530 (2014).

8. A. L. Traud, E. D. Kelsic, P. J. Mucha, M. A. Porter, *SIAM review* **53**, 526 (2011).

9. J. Reichardt, S. Bornholdt, *Physical Review E* **74**, 016110 (2006).

10. S. Fortunato, M. Barthelemy, *Proceedings of the National Academy of Sciences* **104**, 36 (2007).

11. C. Thomas, *et al.*, *Proceedings of the National Academy of Sciences* **111**, 16574 (2014).

12. C. Reveley, *et al.*, *Proceedings of the National Academy of Sciences* **112**, E2820 (2015).

13. O. Sporns, C. J. Honey, R. Kötter, *PloS one* **2**, e1049 (2007).

14. P. Hagmann, *et al.*, *PLoS Biol* **6**, e159 (2008).

15. M. P. van den Heuvel, O. Sporns, *The Journal of neuroscience* **33**, 14489 (2013).

16. D. E. Warren, *et al.*, *Proceedings of the National Academy of Sciences* **111**, 14247 (2014).
